# Assessment of the Performance of the Aptima Bacterial Vaginosis Assay Over a 3-Month Period in a French Hospital

**DOI:** 10.1128/spectrum.01301-22

**Published:** 2022-08-18

**Authors:** L. Ruffier d’Epenoux, E. Tessier, A. Guillouzouic, E. Fayoux, C. Bourigault, P. Bémer, S. Corvec

**Affiliations:** a Service de Bactériologie et des Contrôles Microbiologiques, CHU de Nantes, Nantes, France; b Université de Nantes, CHU Nantes, INSERM, INCIT UMR, Nantes, France; c Service d’Hygiène hospitalière, CHU de Nantes, Nantes, France; University of Utah and ARUP Laboratories

**Keywords:** Nugent scoring, molecular test, *Lactobacillus*, *Gardnerella vaginalis*, *Atopobium vaginae*, bacterial vaginosis

## Abstract

Bacterial vaginosis (BV) is the most common cause of abnormal vaginal discharge. BV represents a dysbiosis with the acquisition of a diverse community of anaerobic bacteria and a reduction in lactobacilli burden. Our objective was to evaluate the Aptima BV assay kit for the diagnosis of BV. From May to August 2019, we enrolled outpatients and inpatients, including nonpregnant women above 18 with vaginosis symptoms, consulting at Nantes University hospital. The Aptima BV assay measures the loads of Gardnerella vaginalis, Atopobium vaginae, and *Lactobacillus* species in relation to overall bacterial load. The Aptima BV assay was compared to Nugent scoring (NS). A total of 456 women were enrolled, and 347 patients met the inclusion criteria with data available for the analysis. NS was used to classify the samples and 144 (41.5%) samples were classified as normal (NS = 0–3), 45 (13%) as BV (NS = 7–10), 38 (11%) presented an intermediate vaginal microbiota (3 < NS < 7), 79 (22.7%) had various bacteria (excluding vaginal flora), 29 (8.3%) had insufficient bacterial density, and 12 (3.5%) had a predominance of yeasts. The Aptima BV kit displayed a sensitivity of 91.1% and specificity of 94.4% with a positive predictive value (PPV) of 83.7% and a negative predictive (NPV) value of 97.1%. The results of this monocentric retrospective study show that Aptima BV kit has a good diagnostic correlation compared to standard of care for dysbiotic diagnosis cases.

**IMPORTANCE** The possibility exists of the involvement of a new molecular test in the routine algorithm of bacterial vaginosis diagnosis in microbiology laboratories. This manuscript reports on our experience, and we propose an organization combining Nugent scoring and molecular testing, especially for intermediate Nugent scores.

## INTRODUCTION

Bacterial vaginosis (BV) corresponds to a vaginal dysbiosis, the main cause of vaginal discharge in women, affecting 29% of the overall population (1–3). In 1955, Gardner and Dukes first defined and described BV. Despite its benign character for nonpregnant women, in pregnant women, BV cause spontaneous abortions, premature births, premature membranes ruptures, or chorioamnionitis (4–6).

BV is characterized by a shift in vaginal flora from the dominant *Lactobacillus* species (which normally represent 95% of the total bacteria of the vaginal flora [7–9]) to a polymicrobial anaerobe-dominated microbiota, including especially Gardnerella vaginalis, Atopobium vaginae, *Mobiluncus* sp., *Prevotella*, *Bacteroides* sp., and *Peptostreptococcus* sp. (3). This modification within the vaginal microbiota is also associated with biochemical and cytological changes, most commonly pathognomonic of BV (10).

In routine clinical practice, BV diagnosis is based on a set of criteria rather than on the detection of a specific causative microorganism. Indeed, BV can be diagnosed clinically by using the Amsel’s criteria (10) and/or by using Nugent scoring (NS) after Gram staining (11). BV is defined by the Amsel’s criteria if three out of the four of the following criteria are met: presence of thin watery homogenous discharge; elevated vaginal pH (>4.5); “fishy” smell either spontaneously or after the addition of 10% potassium hydroxide to vaginal secretions (“whiff test”); and direct microscopic examination revealing vaginal clue cells (exocervical cells lined with small Gram-positive or-negative bacilli) (10, 12, 13). Exploring the cervicovaginal microbiome can also allow the characterization of normal and healthy vaginal ecosystems or modified ones based on their composition. Thus, it has been revealed in 2011 that the vaginal microbiome contains different bacterial communities clustered into five groups: four were dominated by Lactobacillus iners, L. crispatus, L. gasseri, or L. jensenii, whereas the fifth had lower proportions of lactic acid bacteria and higher proportions of strictly anaerobic organisms (14). However, this innovative method remains costly and not available in all laboratories.

Therefore, the NS is the most widely used microbiological method for BV diagnosis in routine clinical practice. After Gram staining, microscopic observation allows for evaluation the presence of lactobacilli, as well as some anaerobic microorganisms such as Gardnerella vaginalis or *Mobiluncus* sp.

With an experienced technologist, Gram staining is more sensitive, whereas the clinical Amsel criteria are more specific. Overall, a concordance of 80% to 90% has been reported (15). In routine clinical practice, the NS is currently the reference test available in around 3 h for BV diagnosis. As a comparison, Amsel’s criteria are based on nonquantifiable, nonreproducible clinical symptoms only (16–19).

Although Gram staining has been widely used for almost 3 decades and is considered the “reference test” of BV diagnosis, this method is not without limitations. For example, this method is often subject to interobserver variability depending on the observer’s skills and experience (20, 21); this technique is also proven to be limited in detecting some species such as *A. vaginae*, *Ureaplasma* spp., and *Mycoplasma* spp.

Recent studies suggest that molecular diagnostic tools would be beneficial to improve BV diagnosis efficiency and that nucleic acid amplification, targeting several BV-associated bacteria, could be performed (22). The Aptima BV assay is an *in vitro* nucleic acid amplification test that uses a real-time transcription-mediated amplification (TMA) for detection and quantitation of rRNA from bacteria associated with BV, including *Lactobacillus* (L. gasseri, L. crispatus, and L. jensenii), Gardnerella vaginalis, and Atopobium vaginae. The assay reports a qualitative result for BV, according to a specific algorithm based on the quantification and ratio of these microorganisms detected or not. However, it does not report results for each microorganism. The assay is intended to help in BV diagnosis using the automated Panther system with dedicated clinician- or patient-collected vaginal swab samples from females with a clinical suspicion or presenting profiles consistent with vaginosis.

This study aimed to assess the performance of the Aptima BV kit on the Panther automation system for the detection of BV through a prospective analysis of a routine clinical practice use of the technique. The experiment was conducted using vaginal samples (VS) taken from nonpregnant women over 18 years of age.

## RESULTS

**FIG 1:**
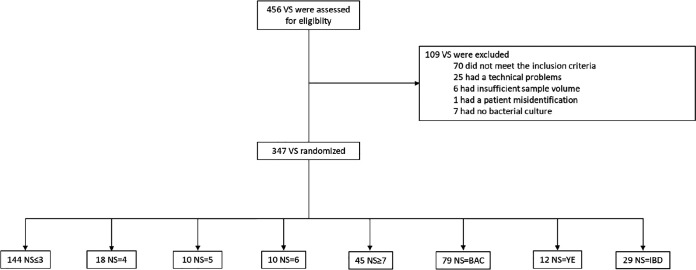
Study flow chart of the vaginal specimens included. NS, Nugent scoring; VS, vaginal samples; BAC, vaginal samples with various bacteria (excluding vaginal flora); YE, vaginal samples with predominance of yeast; IBD, vaginal samples with insufficient bacteria density.

Only specimens meeting the study inclusion criteria were included in the analysis (Fig. 1).

### Characteristics of women enrolled in the study.

A total of 456 women were enrolled in this study; 347 patients met the inclusion criteria with all data available for the analysis. The demographic characteristics associated with these 347 specimens prospectively analyzed in this study are presented in Table 1. Most of the samples (61.7%) were collected from the gynecological emergency ward, from the family planning department, and from hospitalized and outpatients, represented by 6.3%, 7.5%, and 24.5% of the total study population, respectively. According to NS interpretation (positive, negative, or intermediate) after an expert reading by the technician, among the 347 women selected for vaginosis, there were 144 patients (41.5%) with a normal vaginal microbiota (low NS). There were 45 patients (13%) with a positive NS consistent with BV and 38 patients (11%) presenting with an intermediate vaginal microbiota. Finally, 79 patients (22.7%) had various bacteria (excluding vaginal flora), 29 (8.3%) had insufficient bacterial density, and 12 (3.5%) had predominance of yeasts according to NS (Table 1).

**TABLE 1:** The Aptima BV assay validation^*a*^

Patient sample characteristics	Nugent score (0–3) *n* = 144	Nugent score (4–6) *n* = 38	Nugent score (7–10) *n* = 45	BAC *n* = 79	YE *n* = 12	IBD *n* = 29
Median age in yrs; (IQR)	31 (25–38)	33.5 (25.5–37.75)	31 (25–38)	33 (27–38)	29.5 (27–32.25)	35 (32–44)
Hospital type:						
Gynecological emergency	82 (57%)	28 (73.7%)	25 (55.6%)	56 (70.9%)	7 (58.3 %)	16 (64%)
Hospitalised patient	4 (2.8%)	3 (7.9%)	2 (4.4%)	9 (11.4%)	0 (0 %)	8 (32%)
Outpatient	48 (33.3%)	6 (15.8%)	12 (26.7%)	12 (15.2%)	3 (25 %)	4 (16%)
Family planning services	10 (6.9%)	1 (2.6%)	6 (13.3%)	2 (2.5%)	2 (16.7 %)	1 (4%)
Presence of bacterial vaginosis with the vaginosis Aptima BV kit	8 (5.6%)	15 (39%)	41 (91.1%)	23 (29.1 %)	5 (41.7 %)	2 (6.9 %)

a BAC, vaginal samples with various bacteria (excluding vaginal flora); YE, vaginal samples with predominance of yeasts; IBD, vaginal sample with insufficient bacterial density; IQR, interquartile range.

### The Aptima BV assay assessment.

For BV diagnosis, sensitivity, specificity, PPV, and NPV of the Aptima BV test were calculated using all samples (*n* = 189) for which both NS and Aptima BV results were conclusive (either positive or negative) for diagnosis of BV, excluding then the intermediate NS. The Aptima BV test was highly sensitive (91.1%) and specific (94.4%) for diagnosis of BV, with a PPV of 83.7% and a NPV of 97.1% for BV (Table 2).

**TABLE 2:** Test characteristics of the Aptima bacterial vaginosis assay for BV diagnosis

	Aptima bacterial vaginosis assay performance on Panther system			
Reference method: Nugent score (NS)	True positive samples	True negative samples	False positive samples	False negative samples
NS ≤ 3	0	136	8	0
NS ≥ 7	41	0	0	4

A total of 91.1% of NS up to 7 were considered a BV with the Aptima BV test, whereas only 5.6% were also considered positive in the NS less than 3. It is important to note that 39% of intermediate NS was considered BV at the molecular level, which could send a signal in case of doubt (Table 1). It is interesting to note that the higher the NS, the more positive the Aptima BV test is. Indeed, for the 10 NS = 6, 10 NS = 5, and the 18 NS = 4, 70%, 50%, and 16.7%,respectively, were positive with the Aptima BV test.

According to our routine clinical practice organization, the NS was not evaluable for 120 samples. However, when the presence of many bacteria was revealed on direct examination, 29.1% were positive with the Aptima BV test. Coinfection with candidiasis and bacterial dysbiosis was found in almost 42% when yeasts were reported after direct examination. Finally, the Aptima BV test revealed BV in only 6.9% when an insufficient bacterial density on direct examination was observed.

## DISCUSSION

The diagnosis of BV remains a challenge in a microbiology laboratory. Most laboratories still use labor-intensive tests. The reference test (23, 24) still relies on a labor-intensive and time-consuming test performed by experienced technicians (24, 25). This results in consistent difficulties in Gram staining reading. Moreover, despite the formation and the need of a second reading sometimes, the NS may yield inter and intravariability results (26). However, the categorical cutoff value definition allows us to segregate among three groups with an intermediary score between the negative one (score less than 3 = normal flora) and positive one (up to 7 = high probability of bacterial vaginosis). Culture is not always performed routinely. In several cases, the availability of a rapid molecular test may help microbiologists with the diagnosis and clinicians with the management of patients.

Although traditional methods that diagnose BV have relied much more on methods such as the microscopic assessment of bacterial morphotypes (NS) or some combination of patient examination and vaginal discharge (Amsel’s criteria [10]), there exists limited data on molecular detection and BV diagnosis in routine clinical practice and in real life. Although this investigational test is an FDA-approved nucleic acid amplification test for detection of the major causes of bacterial vaginosis, there are limited data on the accuracy of this method (i) to compare with this study, (ii) to assess the relevance of the three-bacteria group detection, and (iii) to implement the routine into clinical practice for a well-defined population.

However, the results of this monocentric retrospective study demonstrate that the Aptima BV assay provides a BV diagnosis with a good correlation and a negative predictive value of 97.1% compared to the NS determination. This new test presents a higher sensitivity compared to previous molecular tests evaluated (24). Indeed, the Aptima BV test assessed during this study presents a 91.1% sensitivity and a 94.4% specificity, whereas the Aptima IVD, BD Affirm, and Hologic ASR revealed 84.4%, 86.7%, and 75.6% sensitivity, and 86.3%, 60.6%, and 81.8% specificity, respectively (24).

Recent research by Frederick et al. has indicated more complex process implicating the role of bacterial pathogens in the etiology of BV (27–29). Many of these organisms, such as Atopobium vaginae, *Prevotella* species, and others, can be detected only by amplification tests (18). Indeed, despite the increasing use of MALDI-TOF technology in microbiology laboratories (with large databases), the identification of these fastidious microorganisms remains challenging, necessitating anaerobic prolonged cultures, not compatible with the delay of results (turnaround time) in the routine clinical practice of BV diagnosis (30). The growth time of these microorganisms is long and some of them are uncultivable (30). Therefore, a molecular assay based on the presence of lactobacilli and the absence of deleterious organisms represents an opportunity to improve the diagnosis of BV in routine clinical practice (28, 31, 32).

Although this new assay performed well, a fuller understanding of its true performance is constrained by the known limitations of the reference methods. Indeed, by using Gram staining, a challenge exists to clearly distinguish Lactobacillus iners (type-III cervicovaginal microbiota) from *G. vaginalis* (33). However, the performance of tests for BV diagnosis could be relevant in the case of specimens with an intermediate NS. In the same way, to limit BV recurrence and optimize the safe and efficacious vaginal probiotic treatment, which reduces the negative effect of antibiotic treatment on other microbiomes or the emergence of antimicrobial resistance (34), this test allows for faster analysis times (3 h on average).

As BV has been significantly associated with preterm labor, premature membrane rupture, preterm delivery, miscarriage, birth asphyxia, low birth weight, and neonatal intensive care unit admission, this rapid molecular detection test may represent an option in early BV screening to prevent adverse maternal and fetal outcomes (35).

This study has limitations. First, as already highlighted in previous studies, the reference test based on the NS with the uncertainty of microscopy reading (24, 25), despite well-trained technicians for BV diagnosis, may explain discrepancies and constitutes a limitation. Second, future studies are needed and should be designed to analyze specific populations of women in order to determine the correlation between the treatment and clinical outcomes, especially in pregnant women. Third, molecular methods are highly specific, which can potentially impact the sensitivity to vaginosis due to minor strains or depending on the initial cervical microbiota. Indeed, this system also does not evaluate the presence of other bacterial strains such as *Prevotella* species, *Megasphera*, *Mobiluncus* species, *Ureaplasma* spp., and Mycoplasma spp. yet associated with BV.

**FIG 2:**
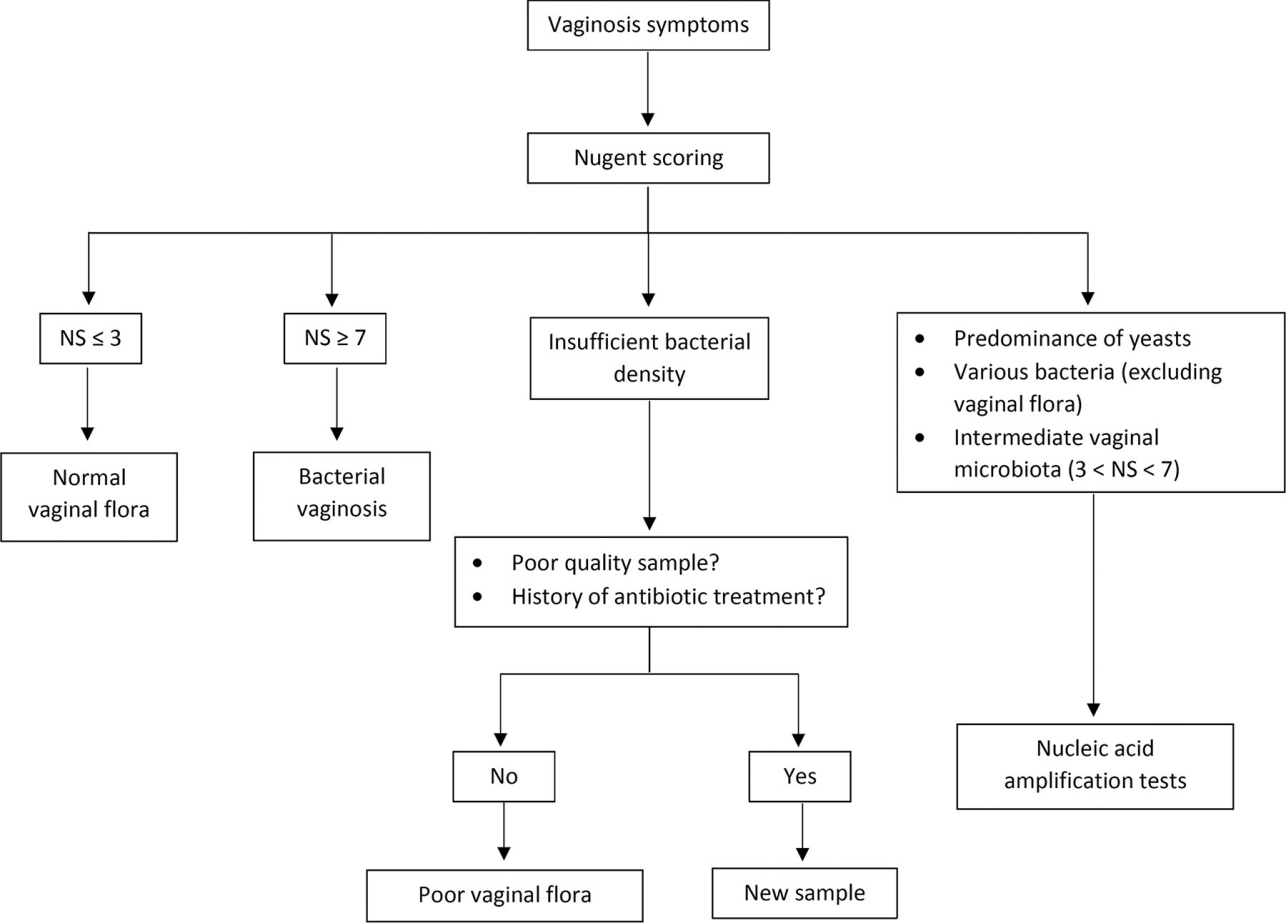
Proposal of an algorithm for routine BV diagnosis. NS, Nugent’s scoring.

To conclude, the availability of a highly sensitive molecular test like the Aptima BV test may improve accurate diagnosis of BV and reduce the delay. In the future, the validation of such multiplexed sample-to-answer tests may change the way patients are managed, and we propose here an algorithm (Fig. 2). Research will be required to demonstrate performance and outcomes in various populations, including pregnant women, asymptomatic women, or women with intermediate NS. In summary, this test appears to be a promising molecular tool for BV routine clinical practice.

## MATERIALS AND METHODS

### Study design.

This study was designed as a monocentric, retrospective, observational study of adult women. From May to August 2019, we enrolled outpatients and inpatients, including nonpregnant women over18 years of age with vaginosis symptoms consulting at Nantes University hospital.

### Ethics approval.

According to the French and European legislation, the use of data in this monocentric study does not need approval from the ethics committee. This study was recorded at Nantes University hospital by the local data privacy officer under the reference: TS005.BIO.AP.2019_14.

Samples for this study, results, and data had been recorded during normal medical care of patients by professionals who are following them. All the data collected for this new study from patient medical folders has been filed in a board under an anonymized code which cannot be tied to the patient.

### Clinical samples.

Vaginal samples (VS) from the patients meeting the inclusion criteria were selected for the study. For the routine clinical practice diagnosis, the vaginal samples (collected using the Copan ESwab in Amies transport medium) for Gram staining and culture, if necessary, was received by the microbiology laboratory. These VS with sufficient volume for Gram staining and standard bacterial culture were also used for the assessment of the Aptima bacterial vaginosis kit on the Panther system. In the laboratory, 200 μL of Copan ESwab collection and transport system was transferred in a tube containing specimen transport media (STM) (Hologic Inc., San Diego, CA, USA) within 24 h of collection and tested immediately on the Panther platform. 

### Vaginal sample comparator testing.

Vaginal samples were tested for BV according to the standard laboratory protocol: NS was performed blind to the molecular test. Gram staining was read by experienced technicians (in case of doubt, a new independent reading was performed). According to Gram staining, (i) a large predominance of Gram-positive bacilli suggesting the presence of *Lactobacillus* spp. from different strains was consistent with a normal vaginal flora (NS between 0–3), (ii) a large predominance of Gram-variable bacilli suggesting the presence of *G. vaginalis* (presence of clue cells), *Mobiluncus* sp., or other anaerobe bacteria with a lack or total absence of *Lactobacillus* spp. was consistent with BV (NS between 7–10), and (iii) an intermediate NS (NS between 4–6) corresponded to potential transition from a normal vaginal flora to a dysbiosis. At Nantes University Hospital, in the microbiology laboratory, a bacterial culture was only performed in four cases: intermediate NS, presence of yeasts on direct examination (YE), insufficient bacterial density on direct examination (IBD) and presence of numerous bacteria on direct examination that did not belong to the vaginal flora (BAC). All media blood (Thermo Fisher, Basingstoke, United Kingdom) and chocolate (Becton, Dickinson, Heidelberg, Germany) agar plates, were incubated at 35 to 37°C in ambient air for 48 h.

### Aptima BV assay.

Each tube containing STM (Hologic Inc., San Diego, CA, USA) was inserted directly into the Panther platform. The Panther system detects and discriminates between four fluorescent signals corresponding to the *Lactobacillu*s group, Atopobium vaginae, Gardnerella vaginalis, and internal control (IC) amplification products. The Panther system software compares signal emergence times for each target microorganism to calibration information to determine either positive BV status, negative BV status, or invalid test of each sample.

An IC is added to each sample. During processing, IC acceptance criteria are automatically verified by the Panther system software. If an IC result is invalid, the sample result is invalidated. Every sample with an invalid IC result must be retested to obtain a valid result.

### Results and discrepant analysis.

Performance of the Aptima BV test was assessed routinely by comparing the results to real life NS scores 0 to 3 (normal vaginal flora) and 7 to 10 (bacterial vaginosis). An Aptima BV result was considered a true positive (TP) or a true negative (TN) only when it matched the result from the comparator method: NS for bacterial culture.

### Calculations and statistical analysis.

Sensitivity was calculated as 100× (no. of TP/[no. of TP + no. of FN]), and specificity was calculated as 100× (no. of TN/[no. of TN + no. of FP]). Positive predictive value was calculated as 100× (no. of TP/[no. of TP + no. of FP]), and negative predictive value was calculated as 100× (no. of TN/[no. of TN + no. of FN]).
